# Accidental Intraoperative Mandibular Fracture in a Third Molar Surgery: When Surgical Skills Are Mandatory in the Face of Empiricism

**DOI:** 10.1155/2023/2263554

**Published:** 2023-07-27

**Authors:** John Nadson Andrade Pinho, Lucas Alves da Mota Santana, Leandro Napier de Souza, Paulo Nand Kumar, Paulo Almeida Júnior, Liane Maciel de Almeida Souza

**Affiliations:** ^1^Department of Dentistry, Federal University of Sergipe (UFS), Aracaju, SE, Brazil; ^2^Department of Oral Surgery and Pathology, School of Dentistry, Federal University of Minas Gerais (UFMG), Belo Horizonte, MG, Brazil; ^3^Department of Oral and Maxillofacial Surgery, Urgency Hospital of Sergipe (HUSE), Aracaju, SE, Brazil

## Abstract

Iatrogenic mandible fractures are rare complications from third molar removal surgeries. While most documented cases stress risk factors inherent to the patient and tooth presentation in fractures' etiology, appreciation of the risk factors underlying the practitioner's skills is scarce. Here, we describe an intraoperative fracture in a healthy 26-year-old female resulting from an incompatible surgical technique during the right mandibular third molar removal. The patient showed facial swelling, pain, malocclusion, and significant mobility of the fractured segment. The surgical management involved an intraoral open reduction with the installation of titanium plates for the fixation of the bone segments. Thus, we highlight that acknowledging the extent of the operator's surgical skills should be part of comprehensive treatment planning, serving as a valuable measure to prevent iatrogenic mandible fractures besides avoiding a traumatic experience for the patient.

## 1. Introduction

Worldwide, third molar removal surgeries integrate many dentists' routines, occasionally resulting in complications. Diverse reasons can be used to indicate third molar extractions, such as caries, mobility, cysts or tumor, periodontal problems, pericoronitis, and preparation for orthognathic surgery [1, 2]. However, complications resulting from these interventions have been described in the literature, ranging from 3.47% to 9.1% second different sources [3, 4]. Among the most frequent complications reported are secondary infection, alveolar osteitis, bleeding, and dysesthesia [5, 6].

Despite the low incidence of complications, a third molar surgery may also lead to a rare, but the most severe of the complications: the mandible fracture [6]. Associated with third molar surgeries, the incidence of mandible fractures is below 0.005% [6, 7]. Different from postoperative fractures, which occur within the first postoperative month, intraoperative fractures—also known as immediate fractures—occur specifically in the transoperatory [7–9].

Together with the clinical evaluation, observing the extent of the operator's skills play a crucial role in comprehensive treatment planning and prevention of mandible fractures [6, 10].

Given the limited number of studies reporting iatrogenic mandible fractures and details of the mismanaged surgeries, the case presented herein aimed to describe an intraoperative fracture resulting from a lower mandibular third molar removal. The lack of comprehensive treatment planning and skills for the occurrence of this injury are also discussed.

## 2. Case Report

**Figure 1:**
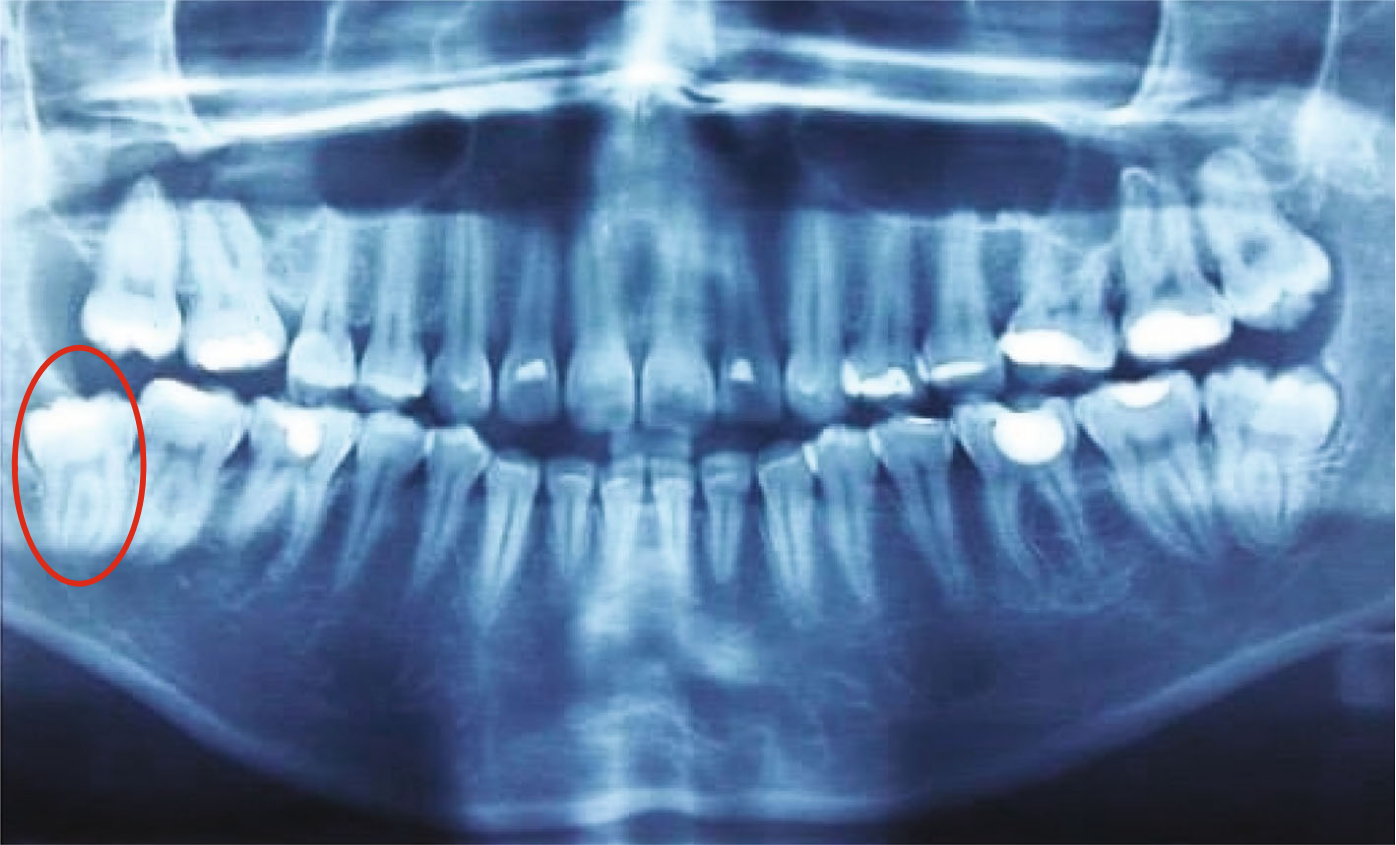
Pre-extraction panoramic radiograph showing tooth #48 characterized by separate roots, distoangulation, and semi-impaction (red circle).

A 26-year-old female was referred by a general practitioner to the Department of Oral and Maxillofacial Surgery of the Urgency Hospital of Sergipe, Brazil, after a traumatic surgical intervention under local anesthesia. During a third molar surgery, the patient noticed a sudden “cracking” noise along with a painful sensation. The previous radiographic analysis revealed the presence of a distoangulated mandibular third molar and B2 position according to Pell and Gregory classification (Figure 1). The audible sound matched an increasing pressure to deliver the tooth. The general practitioner had employed no handpiece before attempting to deliver the third molar.

**Figure 2:**
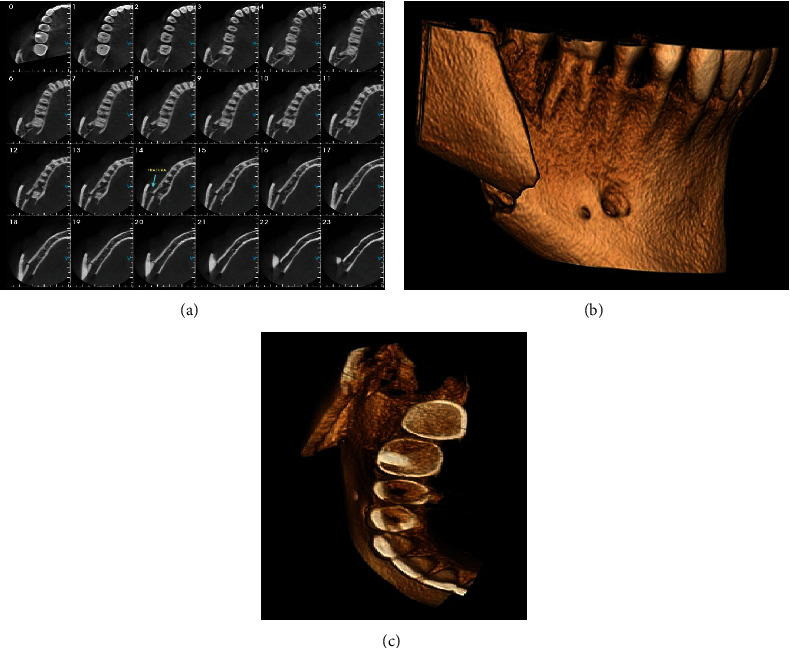
(a) Axial cuts showing right mandibular body fracture. Three-dimensional reconstruction of CT scan showing fracture of the right mandible: (b) lateral view and (c) upper view.

**Figure 3:**
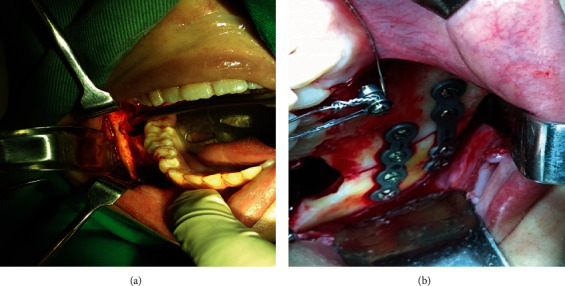
(a) Intraoral approach for fracture reduction. (b) Installation of titanium miniplates for stabilization of bone segments.

Examining the patient in the hospital, besides systemic health, clinical evaluation evidenced intense right-sided facial pain and swelling, palpable step on mandible contour, limited mouth opening, malocclusion, bony mobility of the anterior segment, and the absence of right mandibular third molar. A computed tomography (CT) scan and the 3D reconstruction showed a breach in the continuity of bone through the socket and laterally displaced condylar (distal) fragment related to the mandibular segment in the right-angle site, respectively **(**Figures 2(a), 2(b), and 2(c)). After the resolution of the edema, the mandibular fracture treatment was performed under general anesthesia. The surgeons performed an intraoral open reduction, repositioned the segments, and employed two titanium plates and six-millimeter screws to prevent fragment mobility and ensure primary bone healing **(**Figures 3(a) and 3(b)). The contralateral upper and lower third molars were removed as part of the same surgery.

**Figure 4:**
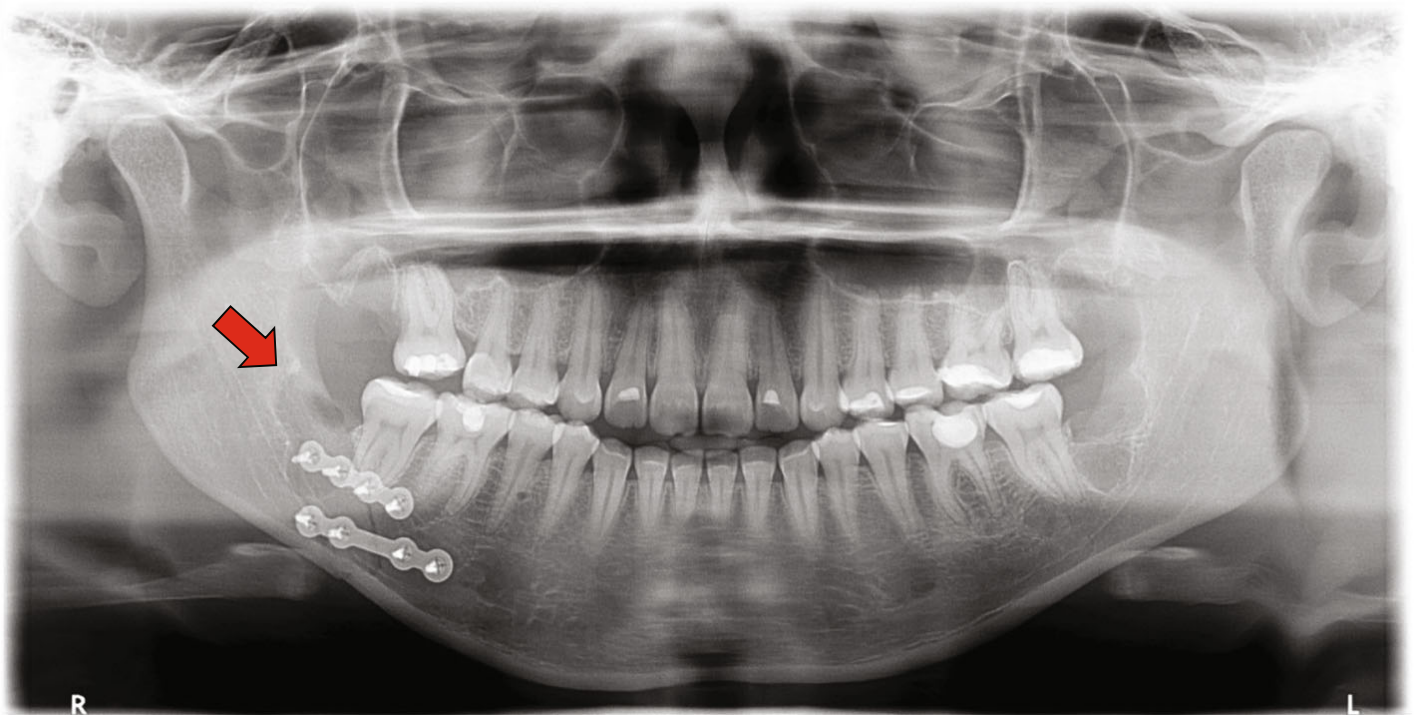
Follow-up panoramic radiograph eight days after the surgery showing reduction and fixation of the fracture (red arrow).

Post-operatively, analgesics and anti-inflammatories were prescribed. In the subsequent months, it was observed satisfactory bone remodeling in the area corresponding to the third molar and restoration of occlusion. The surgical procedure and postoperative recovery were uneventful (Figure 4).

## 3. Discussion

This study addresses an immediate mandible fracture during a wisdom tooth removal surgery. In our report, the general practitioner performed neither osteotomy nor tooth sectioning to remove the B2 and distoangulated tooth [11]. For this reason, these clinical conditions require adequate surgical planning and previous evaluation of potential risks inherent to the procedure, as well as the operator's experience and manual skills.

As patients may end up undergoing hospitalization and major surgeries, a careful assessment to prevent complications is imperative. For instance, some authors have proposed to avoid surgical complications a prophylactic internal fixation guide through dynamic navigation. This technique consists in computer-assisted surgery that guides the correct fixation of an osseointegration plate in an anatomical area with a potential risk of fracture before tooth removal [10]. In addition, comprehensive treatment planning must include the presence of specific risk factors, such as age, sex, dentition, angulation and impaction of the tooth, surgical technique and experience, preoperative infection or bone lesions, and systemic health [6, 8]. In essence, a detailed evaluation helps estimate the surgery's technical expertise and biological cost, conforming them to each patient.

Although the weakening of the mandible due to bone osteotomy or empty alveolus is common ground, postoperative and intraoperative mandibular fractures present distinctive characteristics. Postoperative fractures may be influenced mainly by advanced age, full dentition, and food consistency [12, 13]. In turn, intraoperative fractures may be closely related to poor surgical technique, improper instrumentation, degree of tooth impaction, excessive force, and limited previous radiographic evaluation [6, 8].

In an observational study conducted by Koshy et al. [14], it was demonstrated through the Pell and Gregory model that mandible fractures occur more commonly in distoangular B and A mandibular third molars when compared with other classifications. Besides, this traditional classification is routinely used in dentistry practice for predicting the difficulty level of extracting impacted lower third molars [15]. Undoubtedly, these aspects favor a greater point of weakness of the mandible, especially related to angle fractures. Interestingly, these findings were noted in our case report and may be important predictive factors in the analysis of the potential occurrence of this type of complication.

In contribution, Agrawal et al. [8] described a case of iatrogenic fracture of the mandibular angle during intraoperative of an impacted third molar in a 30-year-old female. Similarly, to our case, the patient had a previous dental history of unsuccessful extraction of the impacted tooth, accompanied by pain, swelling, and malocclusion. Postoperatively, she underwent open reduction and two-point fixation with miniplates. These authors raised some hypotheses that may be of great value for the prevention of this type of complication, such as the presence of bone pathologies, pericoronitis, and periodontal disease.

Of note, even when no intraoperative fracture results from an exaggerated osteotomy—a characteristic of lacking manual dexterity—the bone unnecessarily lost may aggravate the mandible weakening and lead to a postoperative fracture. Despite rare occurrences (~26% of cases), intraoperative fractures of the mandible associated with lower third molar extraction are a potential complication that may impact patients' quality of life, provoke a traumatic experience and tissue injury, such as swelling, pain, facial asymmetry, and nerve damage [6, 7, 16]. Thus, it is mandatory for oral surgeons to ponder the multiple variables involved among different patients to minimize the risks of any complication.

Curiously, most cases are reported in males, mainly between fourth and sixth decades due to the increased bone mineral density and low level of tissue elasticity, narrowing of the periodontal ligament, and ankylosis, which may require more extensive osteotomies [6, 16] and decrease bone resistance, favoring the risk of fractures.

If such a complication occurs, the dental practitioner should duly refer the patient to specialized treatment. Aiming to restore contour, occlusion, and temporomandibular joint function, possible modalities include closed reduction, open reduction, and non-rigid fixation with wire, as well as open reduction and rigid internal fixation with plates or lag screws [17]. Each treatment modality accounts for its reasoning. Regarding rigid internal fixation, for instance, while the single miniplate technique was superior in exhibiting fewer postoperative complications, the two-plate technique was superior in showing more stability [18, 19]. Consequently, weighing the pros and cons of each treatment modality is essential to sound decision-making and individualized therapy for patients based on their conditions.

Finally, an ethical posture is mandatory from the professional involved since iatrogenic mandibular fractures may lead to medicolegal proceedings. After referring the patient to specialized care, the dental practitioner should considerately follow up on the matter until the resolution. In this phase, it has been advocated a soft diet for one month, especially to avoid the possibility of postoperative pathological fractures [16]. In addition, the surgeons, in turn, should understand the distress about the situation and, if possible, treat the patient on priority. The collaborative effort, this way, can soften the inconveniences to the patients and allow their return to the routine.

## 4. Conclusion

One rare but distressful complication that may result from a third molar removal surgery is the mandibular fracture. Besides addressing the risk factors, under no circumstances should dental practitioners neglect to ponder the extent of their surgical skills as a part of comprehensive treatment planning. Based on the case reported, we would, therefore, recommend for the prevention of mandible fractures that dental practitioners prioritize patients' safety and refer them to specialized care in the case of incompatible skills.

## Data Availability

Data supporting this research article are available from the corresponding author or first author on reasonable request.
